# Siglec-1 Is a Novel Dendritic Cell Receptor That Mediates HIV-1 *Trans*-Infection Through Recognition of Viral Membrane Gangliosides

**DOI:** 10.1371/journal.pbio.1001448

**Published:** 2012-12-18

**Authors:** Nuria Izquierdo-Useros, Maier Lorizate, Maria C. Puertas, Maria T. Rodriguez-Plata, Nadine Zangger, Elina Erikson, Maria Pino, Itziar Erkizia, Bärbel Glass, Bonaventura Clotet, Oliver T. Keppler, Amalio Telenti, Hans-Georg Kräusslich, Javier Martinez-Picado

**Affiliations:** 1 AIDS Research Institute IrsiCaixa, Institut d'Investigació en Ciències de la Salut Germans Trias i Pujol, Universitat Autònoma de Barcelona, Badalona, Spain; 2 Department of Infectious Diseases, Virology, Universitätsklinikum Heidelberg, Heidelberg, Germany; 3 Institute of Microbiology, University Hospital Center and University of Lausanne, Lausanne, Switzerland; 4 Institució Catalana de Recerca i Estudis Avançats (ICREA), Barcelona, Spain; Yale University School of Medicine, United States of America

## Abstract

Dendritic cells (DCs) are essential antigen-presenting cells for the induction of immunity against pathogens. However, HIV-1 spread is strongly enhanced in clusters of DCs and CD4^+^ T cells. Uninfected DCs capture HIV-1 and mediate viral transfer to bystander CD4^+^ T cells through a process termed *trans*-infection. Initial studies identified the C-type lectin DC-SIGN as the HIV-1 binding factor on DCs, which interacts with the viral envelope glycoproteins. Upon DC maturation, however, DC-SIGN is down-regulated, while HIV-1 capture and *trans*-infection is strongly enhanced via a glycoprotein-independent capture pathway that recognizes sialyllactose-containing membrane gangliosides. Here we show that the sialic acid-binding Ig-like lectin 1 (Siglec-1, CD169), which is highly expressed on mature DCs, specifically binds HIV-1 and vesicles carrying sialyllactose. Furthermore, Siglec-1 is essential for *trans*-infection by mature DCs. These findings identify Siglec-1 as a key factor for HIV-1 spread via infectious DC/T-cell synapses, highlighting a novel mechanism that mediates HIV-1 dissemination in activated tissues.

## Abstract 2

The novel dendritic cell receptor Siglec-1 binds sialyllactose moieties on HIV-1 membrane gangliosides, thereby enhancing HIV-1 transinfection.

## Author Summary

Mature dendritic cells (mDCs) capture and store infectious HIV-1 and subsequently infect neighboring CD4^+^ T cells in lymphoid organs. This process, known as *trans*-infection, is a key contributor to HIV pathogenesis, but the precise mechanism and the identity of the receptor on the mDC surface that recognizes viral particles remain controversial. Although the interaction of HIV-1 envelope glycoproteins with the C-type lectin DC-SIGN has been suggested to mediate HIV-1 capture and *trans*-infection, later studies revealed an envelope glycoprotein-independent virus capture mechanism in mDCs. Here, we identify Siglec-1 as the surface receptor on mDCs that boosts their uptake of HIV-1 and their capacity to *trans*-infect CD4^+^ cells, leading in turn to HIV-1 disease progression. Siglec-1 captures the virus by interacting with sialyllactose-containing gangliosides exposed on viral membranes. This indicates that Siglec-1 functions as a general binding molecule for any vesicle carrying sialyllactose in its membrane, including exosomes and other viruses. We suggest that this natural pathway through mDC, which would normally lead to antigen processing and presentation, has been subverted by HIV-1 for its own storage and transmission.

## Introduction

HIV-1 can infect CD4^+^ cells of the lymphoid and myeloid lineage with a strong preference for CD4^+^ T cells. Myeloid DCs exhibit innate resistance against HIV-1 infection. HIV-2, on the other hand, efficiently infects myeloid DCs due to its accessory viral protein Vpx, not present in HIV-1, which is able to counteract the myeloid restriction factor SAMHD1 [1],[2]. Despite lacking these additional target cells, HIV-1 exhibits a higher pathogenicity than HIV-2 and has dominated the global AIDS pandemic. Indeed, myeloid DCs can contribute to the spread of HIV-1 through *trans*-infection of CD4^+^ T cells [3],[4]. This process requires HIV-1 binding to the DC surface, viral capture and release of trapped viruses at the infectious synapse, a cell-to-cell contact zone between uninfected DCs and interacting CD4^+^ T cells, which facilitates infection by locally concentrating virus and viral receptors [5].

Classical myeloid DCs patrol submucosal surfaces, where they capture and internalize microbial pathogens through various cell surface receptors. Pioneering studies suggested that HIV-1 is trapped by immature DCs (iDCs) in mucosal tissues through binding of its envelope glycoproteins to the C-type lectin DC-SIGN, with subsequent transfer of infectious particles to secondary lymphoid tissues, where *trans*-infection occurs [4],[6]. Later reports indicated, however, that HIV-1 captured by iDCs is rapidly degraded [7]–[9], arguing against this original “Trojan horse” hypothesis. Conversely, maturation of DCs with lipopolysaccharide (LPS), a microbial product significantly augmented in the plasma of HIV-1-infected individuals [10], markedly enhanced the capacity of DCs to capture HIV-1 and mediate *trans*-infection [5],[7],[9]. These results suggested that HIV-1 capture by LPS-matured mDCs (LPS mDCs) plays an essential role in HIV-1 pathogenesis, facilitating viral spread in the densely populated lymphoid tissue, where many uninfected T cells contact virus-presenting mDCs.

Other receptors besides DC-SIGN have been identified as binding factors for HIV-1 but do not explain why LPS mDC capture of HIV-1 is independent of viral glycoproteins [9]. Instead, HIV-1 capture is markedly sensitive to reductions in viral sphingolipid content [11] and relies on HIV incorporation of membrane gangliosides [12],[13]. Furthermore, we recently showed that sialyllactose in gangliosides serves as the viral attachment factor for LPS mDCs [13]. Since HIV-1 and cellular secreted vesicles, termed exosomes, use the same pathway for mDC capture [11], HIV-1 may have hijacked a pre-existing cellular route for vesicle capture to facilitate efficient transfer to multiple target cells.

## Results

### 1. Siglec-1 Is Up-Regulated in Highly *Trans*-Infecting LPS mDCs

**Figure 1:**
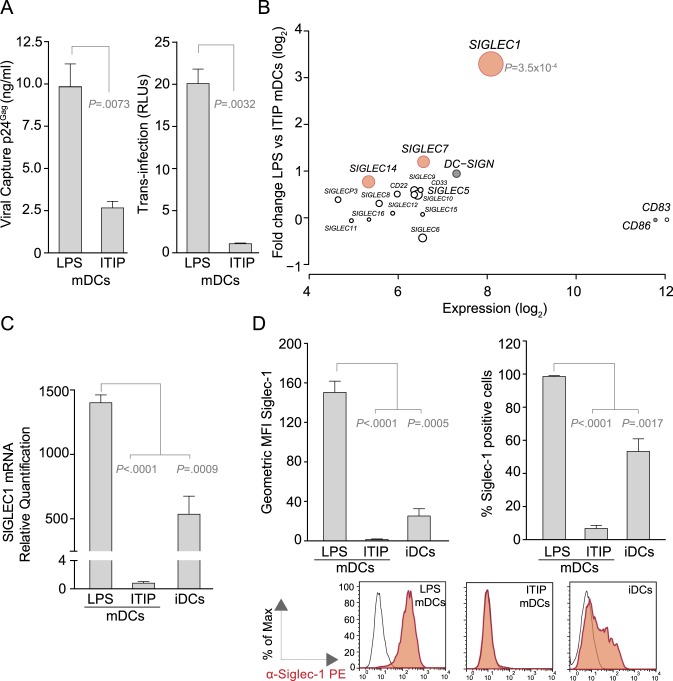
Siglec-1 is up-regulated in highly *trans*-infecting LPS mDCs. (A) (Left) Comparative HIV-1 capture of LPS and ITIP mDCs: cells were cultured with HIV-1, washed, and lysed to measure viral p24^Gag^ antigen by ELISA. (Right) Comparative transmission of captured HIV-1 from LPS and ITIP mDCs to a reporter CD4^+^ cell line. Graphs show mean values and standard error of the means (SEMs) from two independent experiments including cells from six donors. (B) Plot of *SIGLEC* genes (in open circles), *CD86* and *DC-SIGN* (in grey circles) computing the fold change in LPS mDCs compared to ITIP mDCs, and the average gene expression across all samples. Circle size is inversely proportional to adjusted *p* values. Highlighted in red are statistically differentially expressed genes. Analysis was performed with DCs from four donors matured in parallel with the different stimuli. (C) Relative quantification of *SIGLEC1* mRNA expression levels in distinct DCs analyzed by qRT-PCR. Measurements were normalized using the endogenous control housekeeping gene *Beta Glucuronidase*. Data show means and SEMs of samples from six donors. (D) Cell surface expression of Siglec-1 in distinct DCs analyzed by FACS with mAb 7–239-PE. (Left graph) Geometric mean fluorescence intensity (MFI) of Siglec-1. (Right graph) Percentage of Siglec-1 positive cells. Data show mean values and SEM from two experiments, including cells from six donors. (Histograms) Representative profiles of Siglec-1 staining in distinct DCs derived from one donor.

To identify the molecule on DCs that mediates HIV-1 and exosome capture, we performed transcriptome analysis on differentially matured DCs with a highly divergent capacity to capture and transmit HIV-1. We used efficiently *trans*-infecting LPS mDCs and compared them to DCs matured in the presence of the clinical grade cocktail ITIP (ITIP mDCs), which exhibit strongly reduced HIV-1 capture and *trans*-infection capacity (Figure 1A) [14]. We focused our analysis on the Siglec family (including CD83) because these type I transmembrane proteins have an amino-terminal V-set domain that had been shown to interact with sialylated ligands [15]. Most members of the family were equally expressed in LPS mDCs and ITIP mDCs, and this was also observed for the maturation marker CD86 (Figure 1B). *DC-SIGN*, *SIGLEC7*, and *SIGLEC14* were slightly up-regulated in LPS mDCs, but this difference was not statistically significant for *DC-SIGN* and marginally significant for *SIGLEC14* and *SIGLEC7*, respectively (*p* = 0.03 and *p* = 0.04). In contrast, *SIGLEC1* expression was strongly up-regulated in LPS mDCs compared to ITIP mDCs with genome-wide significance (*p* = 3.5×10^−4^; Figure 1B). Furthermore, *SIGLEC1* ranked 20^th^ of all differentially regulated genes in comparative transcriptome analysis. The differential expression of Siglec-1 in LPS and ITIP mDCs was confirmed by quantitative real-time PCR (qRT-PCR; Figure 1C) and Fluorescence Activated Cell Sorting (FACS; Figure 1D). Comparison with iDCs also revealed a significantly higher expression level and surface density of Siglec-1 in LPS mDCs (Figure 1C,D).

### 2. Siglec-1 Expressed in LPS mDCs Capture Distinct Ganglioside Containing Vesicles, Such as HIV-1 Viral-Like Particles, Liposomes, and Exosomes

**Figure 2:**
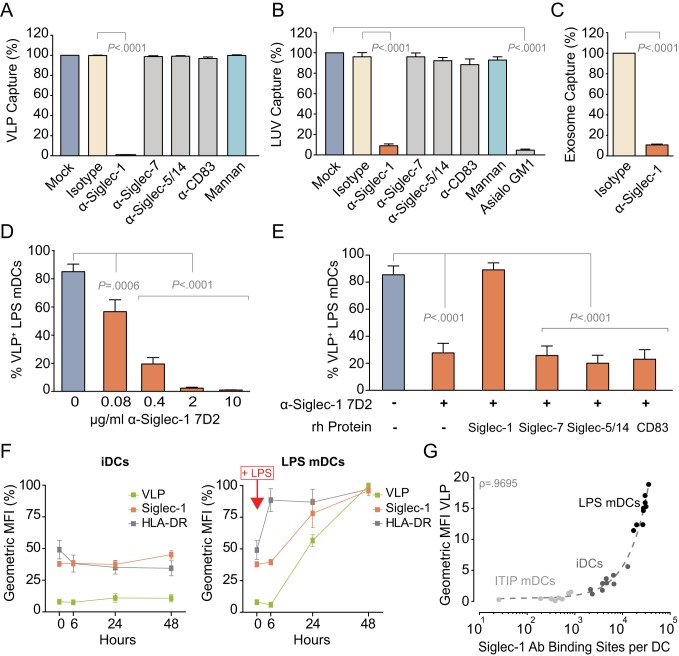
Siglec-1 expressed in LPS mDCs capture distinct ganglioside containing vesicles, such as HIV-1 viral-like particles, liposomes, and exosomes. (A) Relative capture of VLP_HIV-Gag-eGFP_ by LPS mDCs that had been pre-incubated with 10 µg/ml of the indicated mAbs or 500 µg/ml of mannan before VLP exposure for 30 min at 37°C. Values are normalized to the level of VLP capture by mock-treated LPS mDCs (set at 100%). Data show mean values and SEMs from three experiments including cells from nine donors. (B) Relative capture of GM1 containing LUV_HIV-tRed_ by LPS mDCs as described in (A). Data show mean values and SEMs from two experiments including cells from six donors. (C) Relative capture of Exosomes_DiI_ by LPS mDCs that had been pre-incubated with 10 µg/ml of the indicated mAbs before exosome exposure for 4 h at 37°C. Values are normalized to the level of exosome capture by isotype-treated LPS mDCs (set at 100%). Data show mean values and SEMs from two experiments including cells from five donors. (D) Capture of VLP_HIV-Gag-eGFP_ by LPS mDCs that had been pre-incubated with decreasing concentrations of α-Siglec-1 mAb 7D2 before VLP exposure for 30 min at 37°C. Titration of α-Siglec-1 mAb 7–239 is shown in Figure S1. Data show mean values and SEMs from three experiments including cells from six donors. (E) Capture of VLP_HIV-Gag-eGFP_ by LPS mDCs that had been pre-incubated with or without 2 µg/ml of α-Siglec-1 mAb 7D2 previously treated or not with at least a 100-fold molar excess of the indicated human recombinant proteins. Of note, Siglec-14 shares 100% of amino acid homology with Siglec-5 in the V-set domain. Data show mean values and SEMs from three experiments including cells from nine donors. (F) Kinetics of VLP_HIV-Gag-eGFP_ capture by iDCs (left graph) and LPS mDCs (right graph) compared to the expression of Siglec-1 over time, assessed after LPS addition to mDCs. Cells were pulsed for 1 h at 37°C with VLP_HIV-Gag-eGFP_ and labeled for Siglec-1 and HLA-DR in parallel at the indicated time points. For comparative purposes, the maximum geometric MFI values obtained by FACS for each donor were set at 100%. Data show mean values and SEMs including cells from three donors. (G) Positive correlation (ρ = 0.9695) between the geometric MFI of captured VLPs and the mean number of Siglec-1 Ab Binding Sites per cell in different DC subtypes (see also Figure S2 to compare VLP capture capacity among LPS mDCs derived from the same donor). Data show values from three experiments including cells from nine donors.

To test whether Siglec-1 is the surface molecule on LPS mDCs responsible for the capture of vesicles and viruses that carry sialyllactose-containing gangliosides in the outer leaflet of their membrane, we used a previously established FACS assay [11],[13]. This assay makes use of HIV-1 virus-like particles lacking the viral envelope glycoproteins and carrying a fusion of the viral structural protein Gag with eGFP (VLP_HIV-Gag-eGFP_). These fluorescent VLPs follow the same trafficking route as wild-type HIV-1 in LPS mDCs [11]. VLP capture of LPS mDCs was evaluated in the presence of antibodies (Abs) against different Siglecs or mannan, a C-type lectin inhibitor blocking the HIV-1 interaction with DC-SIGN. Besides Siglec-1, we included Abs against CD83, highly expressed in ITIP and LPS mDCs (Figure 1B); Siglec-7, moderately up-regulated in LPS mDCs (Figure 1B); and Siglec-5/14 too, due to their high homology to the V-set domain of Siglec-1. VLP capture was almost completely abolished when LPS mDCs were pre-treated with the α-Siglec-1 monoclonal Ab (mAb) 7D2 (Figure 2A; *p*<0.0001). However, pretreatment with Abs against other Siglec family members or blockade of DC-SIGN with mannan had no effect (Figure 2A).

We have previously shown that Texas Red (tRed) labeled Large Unilamellar Vesicles (LUV) mimicking the size and lipid composition of HIV-1 and containing the ganglioside GM1 (LUV_HIV-tRed_) follow the same trafficking route as VLP_HIV-Gag-eGFP_ in LPS mDCs. Binding and capture in both cases depends on the recognition of sialyllactose exposed in gangliosides of the vesicle membrane [13]. Accordingly, capture of GM1-containing LUV_HIV-tRed_ by LPS mDCs was efficiently and specifically inhibited by the α-Siglec-1 mAb 7D2 (Figure 2B; *p*<0.0001). The residual capture by 7D2-treated LPS mDCs was similar to that exhibited by untreated LPS mDCs capturing LUV_HIV-tRed_ containing GM1 without the sialic acid group (Asialo GM1), confirming that sialic acid in the vesicle membrane is crucial for Siglec-1 recognition (Figure 2B; *p*<0.0001). We extended this analysis to cellular exosomes, which also carry sialyllactose-containing gangliosides in their membrane [16] and can be internalized by LPS mDCs [11]. Fluorescent exosomes were efficiently captured by LPS mDCs, and this capture was almost abolished by mAb 7D2 treatment (Figure 2C; *p*<0.0001).

Titration of the α-Siglec-1 mAb 7D2 revealed a dose-dependent inhibition of VLP capture (Figure 2D). Specificity of the mAb 7D2–mediated inhibition was confirmed by pre-incubation of this mAb with different Siglec proteins. Pre-incubation with purified Siglec-1 completely restored VLP capture, while pre-incubation with purified Siglec-7, -5/14, or CD83 had no effect (Figure 2E). Although the epitope recognized by 7D2 mAb might not constitute the actual viral binding site, since 7D2 Fab fragments did not lead to a block in VLP capture, titration with 7–239, a different α-Siglec-1 mAb, confirmed a dose-dependent inhibition of VLP capture (Figure S1). Hence, α-Siglec-1 mAb 7D2, raised against the four N-terminal domains of Siglec-1 [17], or α-Siglec-1 mAb 7–239, raised against the full 17-domain protein [18], specifically blocked sialyllactose-dependent LPS mDC capture of VLPs, LUVs, and exosomes, identifying Siglec-1 as the relevant recognition receptor.

If Siglec-1 serves as a recognition receptor on DCs, its surface expression should correlate with their respective VLP capture ability. Capture was low in iDCs and stable over time (Figure 2F, left graph), while VLP capture was strongly enhanced following LPS treatment (Figure 2F, right graph). This increased VLP capture ability directly correlated with a strong up-regulation of Siglec-1 surface expression on LPS mDCs (Figure 2F, right graph). We also performed quantitative FACS analysis to determine the absolute number of Siglec-1 Ab Binding Sites (ABS) on ITIP mDCs, iDCs, and LPS mDCs (Figure 2G). The VLP capture capacity of these distinct DC subtypes was strongly correlated with the mean number of Siglec-1 ABS expressed per cell (ρ = 0.9695; Figure 2G). Furthermore, Siglec-1 expression also correlated with the relative VLP capturing capacity of LPS mDCs derived from the same donor (Figure S2). These experiments show a direct correlation between Siglec-1 expression on the DC surface and their respective VLP capture capacity.

### 3. Siglec-1 Captures HIV-1 and Traffics with the Virus to the same Sac-Like Compartment

**Figure 3:**
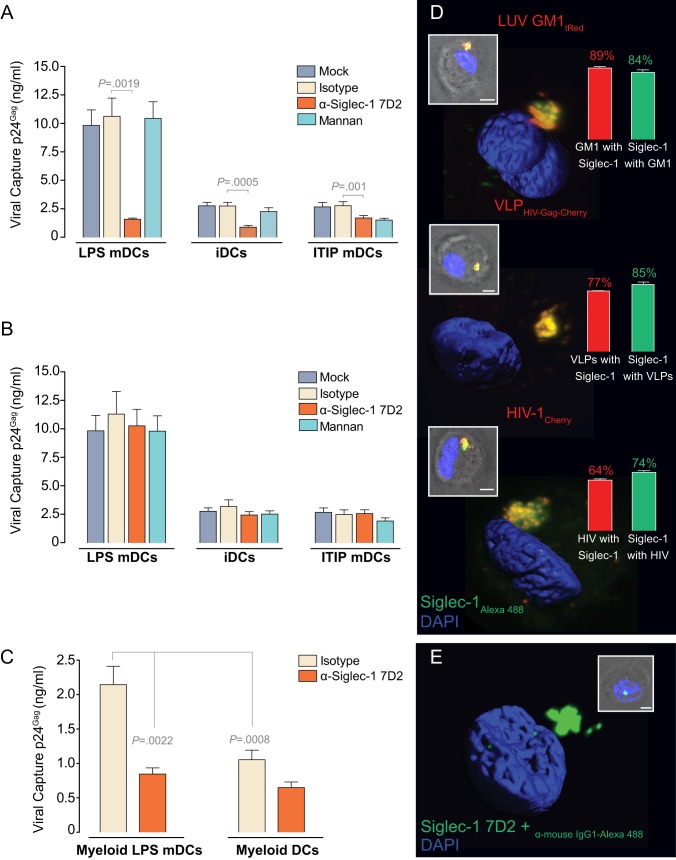
Siglec-1 captures HIV-1 and traffics with the virus to the same sac-like compartment. (A) Comparative capture of HIV-1 by distinct DCs that had been pre-incubated with 10 µg/ml of the indicated mAbs or 500 µg/ml of mannan for 30 min before viral exposure. Cells were cultured with HIV-1 in the presence of the indicated reagents, washed, and lysed to measure p24^Gag^ by ELISA. Viral binding at 4°C in LPS mDCs is shown in Figure S3. Data show mean values and SEMs from two experiments including cells from six donors. (B) Comparative capture of HIV-1 by distinct DCs first exposed to the virus and then treated with the indicated reagents for 30 min before washing. Cells were lysed and assessed by p24^Gag^ ELISA. Data show mean values and SEMs from two experiments including cells from six donors. (C) Comparative capture of HIV-1 by distinct blood myeloid DCs that had been pre-incubated with 10 µg/ml of the indicated mAbs for 30 min before viral exposure as in panel A. Figure S4 depicts Siglec-1 surface expression levels of blood myeloid cells. Data show mean values and SEMs from two experiments including cells from six donors. (D) Confocal microscopy analysis of LPS mDCs pulsed for 4 h with GM1-containing LUV_HIV-tRed_, VLP_HIV-Gag-Cherry_ or HIV-1_Cherry_, fixed, permeabilized, and then stained for Siglec-1 with mAb 7–239-Alexa 488. (Inset) Merge of the bright field and maximun fluorescence intensity (scale bar: 5 µm). (3D images) Isosurface representation of DAPI stained nucleus and maximum fluorescence intensity of the sac-like compartment where particles and Siglec-1 accumulate are shown in a 3D volumetric x-y-z data field. (Bar graphs) Quantification of the percentage of GM1-containing LUV_HIV-tRed_, VLP_HIV-Gag-Cherry_ or HIV-1_Cherry_ co-localizing with Siglec-1-Alexa 488 7–239 and vice versa, obtained analyzing at least 50 compartments from LPS mDCs of two donors. The mean and standard deviation of the thresholded correlation coefficient of Pearson (obtained considering all the images) were 0.77±0.07, indicating co-localization. See also Movies S1, S2, S3 or Figure S5 to observe the compartment in relation to the plasma membrane or the cytoplasm of the cells. (E) Confocal microscopy analysis showing the sac-like compartment pattern of Siglec-1 in LPS mDCs after internalization of the α-Siglec-1 mAb 7D2. Cells were labeled with the mAb for 30 min at 16°C, revealed with an Alexa 488 secondary Ab, shifted to 37°C for 4 h, and analyzed. (3D image) 3D reconstruction (representative of 69% of the analyzed DCs) was done as in (D). (Inset) Merge of the bright field and maximun fluorescence intensity (scale bar: 5 µm).

To extend these observations to authentic virus, we performed similar experiments with infectious HIV-1. Again, LPS mDCs captured significantly more virus than iDCs or ITIP mDCs (Figure 3A). The α-Siglec-1 mAb 7D2 inhibited HIV-1 capture of LPS mDCs by 80% (Figure 3A; *p* = 0.0019), while pre-treatment with mannan had no effect. Noteworthy, α-Siglec-1 mAbs also blocked binding of HIV-1 to LPS mDCs at 4°C (Figure S3). Similarly, pre-treatment of iDCs with the mAb 7D2 reduced HIV-1 capture by 60% (Figure 3A; *p* = 0.0005), indicating that even at lower surface expression levels of Siglec-1 on iDCs (Figure 1D), this receptor still constitutes an important capture moiety. Consistently, capture inhibition by mAb 7D2 was much weaker on ITIP mDCs (Figure 3A; *p* = 0.001), which exhibited the lowest Siglec-1 surface expression (Figure 1D). The effect of the mAb 7D2 on HIV-1 capture was dependent on blocking cell surface Siglec-1, as addition of the inhibitor after virus exposure had no effect (Figure 3B). Importantly, primary blood myeloid DCs exposed to LPS also up-regulated Siglec-1 expression levels (Figure S4) and showed increased HIV-1 capture capacity that could be blocked by pretreatment with α-Siglec-1 mAb 7D2 (Figure 3C; *p* = 0.0022). These results strongly suggest that Siglec-1 is the molecule responsible for HIV-1 capture by DCs, especially upon triggering of Siglec-1 expression by LPS.

Next, we investigated whether Siglec-1 traffics together with sialylated ligands, such as ganglioside-containing liposomes, VLPs, or HIV-1, reaching the same sac-like compartment where these particles are stored [11],[13]. LPS mDCs were pulsed with these different fluorescent particles and subsequently stained with the α-Siglec-1 Alexa 488 mAb 7–239 (Figure 3D). Confocal microscopy revealed extensive co-localization of Siglec-1 with GM1-containing LUV_HIV-tRed_, VLP_HIV-Gag-Cherry_, and HIV-1_Cherry_ in the same compartment (Figure 3D, Movies S1, S2, S3, and Figure S5). We then assessed whether binding of α-Siglec-1 mAb 7D2 to LPS mDCs would be sufficient to internalize Siglec-1 into a similar compartment. Following incubation for 4 h at 37°C, most of the bound α-Siglec-1 mAb 7D2 was indeed found within a sac-like compartment (Figure 3E). Hence, binding of mAb 7D2, probably causing Siglec-1 cross-linking at the cell surface, is sufficient to induce Siglec-1 internalization.

### 4. Siglec-1 Mediates HIV-1 *Trans*-Infection to Target Cells and Accumulates at the Infectious Synapse

**Figure 4:**
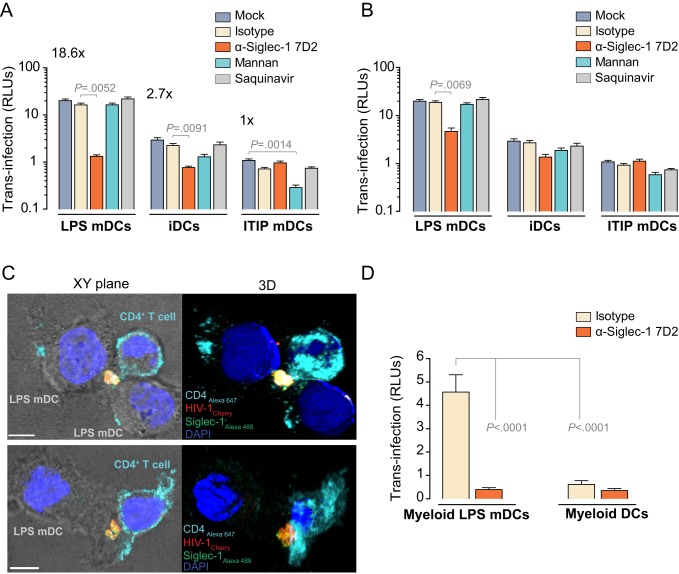
Siglec-1 mediates HIV-1 *trans*-infection to target cells and accumulates at the infectious synapse. (A) HIV-1 transmission from distinct DCs to a reporter CD4^+^ cell line. DCs were pre-incubated as in Figure 3A, washed, and co-cultured with reporter cells for 48 h. HIV-1 infection of reporter cells was determined by induced luciferase activity in relative light units (RLUs). Data show mean values and SEMs from two experiments including cells from six donors. (B) HIV-1 transmission from distinct DCs first exposed to the virus and then treated with the indicated reagents for 30 min before washing and co-culture with reporter cells. Data show mean values and SEMs from two experiments including cells from six donors. (C) Confocal microscopy analysis of LPS mDCs pulsed with HIV-1_Cherry_ and then co-cultured with CD4^+^ T cells to reveal Siglec-1 localization. Co-cultures were stained with α-CD4-Alexa 647 mAb to identify the membrane of CD4^+^ T cells, fixed, permeabilized, and labeled with α-Siglec-1-Alexa 488 7–239 mAb. (Left images) Merge of the bright field and the fluorescence of an x-y plane (scale bar: 5 µm). (Right images) Isosurface representation of DAPI-stained nucleus and maximum fluorescence intensity of the compartment where HIV-1_Cherry_ and Siglec-1 accumulate in the contact zone with a CD4^+^ T cell, shown in a 3D volumetric x-y-z data field. (D) HIV-1 transmission to reporter cells from distinct blood myeloid DCs that had been pre-incubated with 10 µg/ml of the indicated mAbs for 30 min before viral exposure as in (A). Data show mean values and SEMs from three experiments including cells from twelve donors.

To assess the relevance of Siglec-1 for HIV-1 *trans*-infection, we pulsed distinct DCs with equal amounts of infectious virus in the presence or absence of blocking reagents and cocultured them with a CD4^+^ reporter cell line (Figure 4A). Controls performed with the protease inhibitor saquinavir, which abolishes production of infectious virus, demonstrated that this assay measured only *trans*-infection of reporter cells by DC-captured virus without a contribution from potentially de novo infected DCs (Figure 4A–B, last bars). Pretreatment of LPS mDCs with the α-Siglec-1 mAb 7D2 inhibited HIV-1 *trans*-infection by 85% (Figure 4A; *p* = 0.0052), while blocking DC-SIGN through mannan had no effect. Analogously, pretreatment of iDCs with 7D2 reduced HIV-1 *trans*-infection by 55% (Figure 4A; *p* = 0.0091). In contrast, ITIP mDC-mediated *trans*-infection was not affected by 7D2 but was blocked by mannan (Figure 4A; *p* = 0.0014). Addition of any of the inhibitors tested after DC viral pulse had no significant effect on *trans*-infection (Figure 4B), except for the mAb 7D2 in LPS mDCs (*p* = 0.0069). This latter inhibitory effect could not be explained by differences in viral capture (Figure 3B) but is most likely attributed to the cell-to-cell adhesion function of Siglec-1 [19], where establishment of infectious synapses may be partially impaired when Siglec-1 is blocked in LPS mDCs. Indeed, when we analyzed infectious synapse formation between HIV-1_Cherry_ pulsed LPS mDCs cocultured with CD4^+^ T cells, Siglec-1 polarized towards the site of the cell-to-cell contact zone where viruses were also concentrated (Figure 4C). The importance of Siglec-1 for HIV-1 *trans*-infection was also confirmed for blood myeloid DCs. LPS stimulation strongly enhanced their potential for *trans*-infection (Figure 4D; *p*<0.0001), and this increase could be abolished by pre-incubation with mAb 7D2 (Figure 4D; *p*<0.0001).

### 5. SIGLEC1 Silencing Blocks Viral Capture and *Trans*-Infection, While De Novo Expression of SIGLEC1 Rescues It

**Figure 5:**
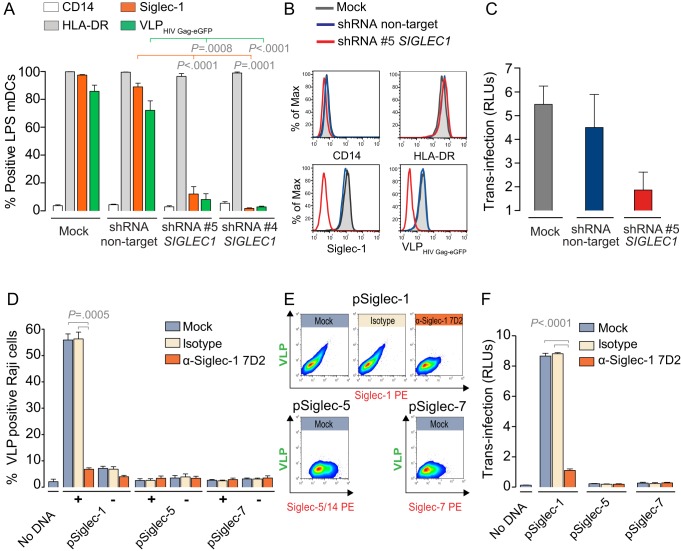
*SIGLEC1* silencing blocks viral capture and *trans*-infection, while de novo expression of *SIGLEC1* rescues it. (A) Interference of *SIGLEC1*. Percentage of LPS mDCs positive for CD14, HLA-DR, Siglec-1, or VLP capture following mock transduction or transduction with nontarget or two different *SIGLEC1*-specific shRNAs. Data show mean values and SEMs from four experiments including cells from at least four donors. (B) Representative cell surface expression levels of CD14, HLA-DR, or Siglec-1 and VLP_HIV-Gag-eGFP_ capture profile of LPS mDCs transduced with nontarget shRNA (blue), *SIGLEC1* target shRNA (red), or mock transduced (grey). (C) HIV-1 transmission to CD4^+^ reporter cells of LPS mDCs that had been mock-transduced or transduced with nontarget or *SIGLEC1*-specific shRNA. DCs were pulsed with HIV-1, washed, and co-cultured with reporter cells for 48 h. HIV-1 infection of CD4^+^ reporter cells was determined by induced luciferase activity in RLUs. Data show mean values and SEMs from two experiments including cells from four donors. (D) Transfection of Siglecs in Raji B cells (see also Figure S6 for transfection efficiencies). Capture of VLP_HIV-Gag-eGFP_ by Raji cells transfected with the indicated expression plasmids for Siglecs or mock transfected. Transfected Raji cells were pre-incubated with 10 µg/ml of the indicated mAbs and exposed to VLPs. See Figure S7 for blocking effect of sialyllactose. Data show mean values and SEMs from two experiments including cells from four transfections. Figure S8 shows results for Siglec transfections in HEK-293T. (E) Representative dot plots from Siglec-1, Siglec-5, and Siglec-7 transfected Raji cells pre-incubated with the indicated mAbs and subjected to VLP capture. (F) HIV-1 transmission from Raji cells transfected with the indicated expression plasmids for Siglecs to reporter CD4^+^ cells. Transfected cells were pre-incubated with the indicated mAbs as in (C) and then exposed to HIV-1. Data show mean values and SEMs from two experiments including cells from four transfections. Figure S9 depicts viral capture and transmission of viruses with or without the envelope glycoproteins.

To verify the essential role of Siglec-1 during HIV-1 capture and *trans*-infection, we applied two complementary experimental strategies: RNA interference to reduce Siglec-1 expression levels in LPS mDCs and transfection of Siglec-1 into cells devoid of this receptor. In the first approach, we transduced DCs with lentiviral particles coding for different shRNAs by co-infection with vpx-expressing lentiviruses to counteract the restriction factor SAMHD1 and facilitate DC productive infection. Transduction of two different *SIGLEC1*-specific shRNAs, but not of a nontarget shRNA control, led to a drastic decrease in Siglec-1 surface expression and a concurrent loss of VLP_HIV-Gag-eGFP_ capture (Figure 5A,B). Furthermore, transduction of a *SIGLEC1*-specific shRNA, but not of a control shRNA, decreased LPS mDC capacity for HIV-1 *trans*-infection to a reporter CD4^+^ cell line (Figure 5C). We next assessed whether endogenous Siglec-1 expression in cells lacking this molecule on their surface could rescue their capacity for HIV-1 capture and *trans*-ifection. This was first attempted for the monocytic cell line THP-1, but could not be pursued since transfection with any of the plasmids tested up-regulated Siglec-1 expression, probably through TLR signaling (Figure S6A, top panels). Thus, we chose Raji B cell line instead, which lack endogenous expression of Siglec-1 and could be efficiently transfected without unspecific up-regulation of Siglec-1 (Figure S6A, bottom panels, and S6B). Transfection of a Siglec-1 expression vector significantly enhanced VLP_HIV-Gag-eGFP_ capture in the Siglec-1-positive cell population, and this effect was abolished by pretreatment with the α-Siglec-1 mAb 7D2 (*p* = 0.0005; Figure 5D,E). No increased capture was seen in the Siglec-1-negative population of Siglec-1 transfected cells or following transfection of Siglec-5 or Siglec-7 expression plasmids (Figure 5D,E). Pre-incubation with sialyllactose also blocked VLP capture in Siglec-1 transfected Raji cells (Figure S7). Accordingly, transfection of a Siglec-1 expression vector into Raji cells significantly increased their capacity for HIV-1 *trans*-infection to a reporter CD4^+^ cell line (Figure 5F), and this effect was again abolished by pre-incubation of transfected cells with the mAb 7D2 (*p*<0.0001; Figure 5F). Equivalent results were obtained when Siglec-1 transfected HEK-293T cells were analyzed (Figure S8). We finally verified that as opposed to DC-SIGN, Siglec-1 viral capture does not rely on the recognition of envelope glycoproteins (Figure S9). Transfection of a Siglec-1 expression vector in Raji cells allowed for efficient capture of HIV-1 with or without envelope glycoproteins, whereas Raji DC-SIGN cells only captured viruses bearing glycoproteins (Figure S9). The complementary approaches of *SIGLEC1* knockdown and de novo expression on heterologous cells strongly support our conclusion that Siglec-1 is a central molecule mediating HIV-1 capture and *trans*-infection.

## Discussion

Three lines of evidence identify Siglec-1 as a novel DC receptor for HIV-1 capture and *trans*-infection: (i) Siglec-1 expression correlates with viral capture and *trans*-infection capacity of DCs, (ii) mAbs against Siglec-1 specifically inhibit HIV-1 capture in a dose-dependent manner, and (iii) *SIGLEC1* knockdown reduces viral capture and *trans*-infection, while heterologous de novo expression of Siglec-1 enhances HIV-1 capture and *trans*-infection. An important role for Siglec-1 in HIV-1 infection is in line with previous studies reporting increased expression of Siglec-1 on CD14^+^ monocytes and macrophages in HIV-1 infection [20]–[22]. However, these studies analyzed Siglec-1 interactions with sialylated viral envelope proteins, while our results clearly show that HIV-1 capture depends on sialyllactose on viral membrane gangliosides interacting with Siglec-1, but does not require viral glycoproteins.

DC-SIGN was initially proposed as the HIV-1 attachment factor concentrating virus particles on the surface of DCs [4], but later studies showed a variable contribution of DC-SIGN to HIV-1 capture and *trans*-infection [23]. Our results indicate that both DC-SIGN and Siglec-1 contribute to *trans*-infection by iDCs, while HIV-1 capture by highly *trans*-infecting LPS mDCs is independent of DC-SIGN and requires Siglec-1. Hence, although Siglec-1 viral binding via sialyllactose recognition does not discriminate between infectious or noninfectious HIV-1 particles, the greater the expression of Siglec-1, the greater the amount of virions captured and transmitted by DCs, diminishing the relative contribution of DC-SIGN gp120-mediated viral capture to *trans*-infection. Given that lectins such as DC-SIGN and Siglec-1 generally achieve high-avidity binding by clustering of both receptor and ligand [15], recognition of thousands of sialyllactose containing gangliosides in the viral membrane by Siglec-1 should be clearly superior to the interaction of DC-SIGN with only 14±7 envelope trimers per virion [24]. Siglec-1 is the only Siglec family member tested that mediated HIV-1 capture, although all Siglecs interact with sialic acid through their respective V-set domains. This could be caused by different specificities, but is most likely due to Siglec-1 containing the largest number of Ig-like C2-type domains of all Siglecs; these domains act as spacers separating the ligand-binding site from the cell surface. Therefore, Siglec-1 extends beyond the glycocalix of the cell, and is thus available for interaction with external ligands, while other family members mainly bind ligands in *cis* [15].

Although Siglec-1 expression is restricted to myeloid cells, particularly to tissue macrophages found in secondary lymphoid tissues [17],[25], its expression can be rapidly induced and up-regulated once myeloid cells are activated [26]. Indeed, DCs exhibit a characteristic mature phenotype in HIV-1 viremic patients [27], and up-regulation of Siglec-1 on mDCs is therefore likely to play an important role in HIV-1 dissemination in lymphoid tissues, thus contributing to HIV-1 disease progression. DC maturation is probably not directly induced by HIV-1 [28], but is more likely a consequence of factors released upon HIV-1 infection. Circulating LPS has been shown to be significantly augmented in HIV-1 patients due to the increased translocation of microbial products from the gastrointestinal lumen once infection is established [10]. Thus, LPS may facilitate HIV-1 progression by local and systemic stimulation of DCs, which (i) leads to Siglec-1 up-regulation and enhanced viral spread and (ii) creates the pro-inflammatory milieu associated with HIV-1 infection and immune activation.

This work together with several other recent reports indicates that HIV-1 uses a highly sophisticated strategy to evade DC immune surveillance and facilitate disease progression. Viral capture through Siglec-1 on the mDC surface is beneficial for viral spread through *trans*-infection, but could also be detrimental for the virus if leading to successful antigen presentation. However, captured HIV-1 do not appear to reach the endolysosomal compartment of LPS mDCs [29], where antigen processing occurs. Furthermore, interaction of HIV-1 with DC-SIGN can cause down-regulation of MHC class II molecules and interferon genes, impairing antiviral immune responses while triggering infectious synapse formation [30]. If productive fusion of the viral and cellular membrane occurs, HIV-1 replication is blocked by the myeloid-specific restriction factor SAMHD1 [1],[2], thus preventing viral antigen production. On the other hand, if DC resistance to infection is bypassed, the interaction of newly synthesized HIV-1 proteins with a cell-intrinsic sensor elicits antiviral immune responses, not typically engaged owing to the absence of DC infection [31].

Siglec-1 captures HIV-1 through its interaction with sialyllactose-containing gangliosides exposed on viral membranes, and therefore functions as a general recognition receptor for vesicles carrying sialyllactose in their membrane. These include exosomes [16] and probably other sialyllactose-containing viruses. Gangliosides have been observed in the membrane of, for example, vesicular stomatitis virus (VSV), semliki forest virus, or murine leukemia virus [32],[33], but are likely to be present in the membrane of many other enveloped viruses as well. Siglec-1-dependent viral capture may be important for direct infection of DCs in these cases, but may also enhance immune recognition, thus benefiting the host. Accordingly, Siglec-1 has been shown to efficiently capture VSV in vivo and facilitate antiviral responses and prevent viral neuroinvasion [34],[35]. The observation that Siglec-1 also captures cellular microvesicles suggests that this pathway normally leads to antigen presentation through exosomes [36] and has been hijacked by HIV-1 for infectious virus storage and spread. The discovery of the role of Siglec-1 in capturing sialylated viruses expands our understanding of HIV-1 transmission mechanisms and warrants novel therapeutic approaches aimed to prevent viral dissemination.

## Materials and Methods

### Ethics Statement

The institutional review board on biomedical research from Hospital Germans Trias i Pujol approved this study.

### Primary Cell Cultures

Peripheral blood mononuclear cells (PBMCs) were obtained from HIV-1-seronegative donors, and monocyte populations or myeloid DCs were isolated and cultured as described in [9]. Monocyte-derived mature DCs were differentiated for 48 h with 100 ng/ml of LPS (Sigma-Aldrich) or ITIP (300 IU/ml IL-1β, 1,000 IU/ml IL-6, 1,000 IU/ml TNF-α, all from CellGenix, and 1 µg/ml PGE2 from Sigma-Aldrich). LPS myeloid DCs were differentiated for 24 h with 100 ng/ml of LPS. Autologous and heterologous CD4^+^ T cells were enriched from PBMCs using the RossetteSep α-CD8^+^ cocktail (Stem cell) and maintained in RPM1 with 10% fetal bovine serum (FBS, Invitrogen) supplemented with 10 IU/ml of IL-2 (Roche).

### Transcriptome Analysis

DCs (3×10^6^) were centrifuged and resuspended in RNAlater solution (Ambion). After lysate homogenization using QIAshredder spin columns (Qiagen), total RNA isolation was performed with the RNeasy Mini Kit (Qiagen), including a 15-min DNAse I treatment step. Affymetrix GeneChip Human Gene 1.0 ST arrays were processed with R using affy and limma Bioconductor packages [37],[38]. After robust multichip average and quantile normalisation, differential expression was computed using moderated paired *t* test. Adjusted *p* values were computed with the Benjamini & Hochberg method [39], and a 0.05 cutoff was applied to select significant genes.

### Comparative Gene Expression Analysis by qRT-PCR

In total, 1 µg of RNA obtained as in the previous section was reverse transcribed using the TaqMan reverse transcription reagents (including multiscribe reverse transcriptase and random hexamers; Applied Biosystems). Predesigned TaqMan gene expression assays and the comparative Ct (ΔΔCt) method [40] were used to determine relative *SIGLEC1* gene expression. *SIGLEC1* mRNA quantification (FAM dye-labeled probe) was normalized using the endogenous control gene *Beta Glucuronidase* (VIC/TAMRA dye labeled probe) in multiplex qPCR experiments run on the Applied Biosystems 7500/7500 Fast Real-Time PCR System and analyzed with the 7500 Software v2.0.4. A cDNA sample from PBMCs was used as a reference for all relative quantification results.

### Siglec-1 Surface Expression Analysis by FACS

DCs were blocked with 1 mg/ml of human IgG (Baxter, Hyland Immuno) and stained with α-Siglec-1-PE 7–239 mAb (AbD Serotec) following the manufacturer's instructions at 4°C for 20 min. Samples were analyzed with FACSCalibur (Becton-Dickinson) using CellQuest and FlowJo software to evaluate collected data.

### Cell Lines, Plasmids, and Viral Stocks

HEK-293T and TZM-bl (obtained through the U.S. National Institutes of Health [NIH] AIDS Research and Reference Reagent Program, from JC Kappes, X Wu, and Tranzyme Inc.) were maintained in D-MEM (Invitrogen). Raji B cell line (kindly provided by Y. van Kooyk) was cultured in RPMI (Invitrogen). Raji DC-SIGN B cell line (kindly provided by Y. van Kooyk) was maintained in RPMI with 1 mg/ml of G418 (Invitrogen). All media contained 10% FBS, 100 IU/ml of penicillin, and 100 µg/ml of streptomycin (all from Invitrogen). VLP_HIV-Gag-eGFP_ and VLP_HIV-Gag-Cherry_ were obtained as previously described [11]. HIV_NL4-3_ was obtained following transfection of the molecular clone pNL4-3 (NIH AIDS Research and Reference Reagent Program from M. Martin). HIV_NL4-3-Cherry_ was obtained following cotransfection of pCHIV and pCHIV mCherry in a 1∶1 ratio [41]. HIV_NL4-3_ lacking the envelope glycoprotein was obtained as described elsewhere [9]. The p24^Gag^ content of the viral stocks and VLP was determined by ELISA (Perkin-Elmer) or by a quantitative Western blot [13]. HIV_NL4-3_ used in infectious assays was titrated employing the TZM-bl reporter cell line as described in [42].

### Production of Liposomes and Exosomes

Large unilamellar vesicles (LUVs) were prepared as in [13], and exosomes were isolated from Jurkat cells as described in [11].

### VLP, Liposome, Exosome, and HIV-1 Capture Assays

LPS mDCs (2×10^5^) were pre-incubated at 16°C for 30 min with 10 µg/ml of α-Siglec-1 mAb 7D2 (HSn 7D2, Abcam), IgG1 isotype control mAb (107.3, BD Bioscience), α-Siglec-7 cell-adhesion neutralizing pAb (R&D Systems), α-Siglec-5/14 cell-adhesion neutralizing mAb (194128; R&D Systems, which recognizes both Siglec-5 and Siglec-14, sharing 99% of amino acid homology in the three extracellular distal domains) or α-CD83 mAb (HB15e; R&D Systems) or with 500 µg/ml of mannan from Saccharomyces cerevisiae (Sigma-Aldrich). Capture experiments were performed maintaining compound concentration and pulsing mDCs in parallel applying either 200 µM of the respective LUV_HIV-tRed_ formulations or 150 ng of VLP_HIV-Gag-eGFP_ Gag per 2×10^5^ cells for 30 min at 37°C. Exosome_DiI_ capture was performed pulsing 1×10^5^ pretreated LPS mDCs with 150–250 µg of exosomes for 4 h at 37°C. After extensive washing, positive DCs were acquired by FACS. To test for potential cross-reactivity of α-Siglec-1 mAb 7D2, 2.2 µM of the mAb were pre-incubated or not with more than 100-fold molar excess of recombinant human protein Siglec-1, and more than 200-fold molar excess of Siglec-7, Siglec-5/14, or CD83 (all from R&D Systems) 30 min at RT prior addition to the LPS mDCs. After incubation with mixes, LPS mDCs were pulsed with VLPs as indicated earlier. Fab fragments were generated from α-Siglec-1 7D2 and Isotype mAbs using the Fab Micro Preparation kit (Pierce) according to the manufacturer's instructions. Quality of Fab preparations was assessed with SDS-PAGE and Coomassie staining. Titration of a different α-Siglec-1 mAb was performed with functional grade clone 7–239 (AbD Serotec). DCs were also assessed for VLP capture for 1 h as described above but starting 5 d after isolation (when LPS was added to LPS mDCs) and continuing 6, 24, and 48 h after LPS addition. In parallel, DCs were labeled with α-Siglec-1-PE 7–239 mAb and α-HLA-DR-PerCP (clone L243, BD Biosciences). The mean number of Siglec-1 Ab binding sites per cell was obtained with a Quantibrite kit (Becton Dickinson) at day 7 as previously described for DC-SIGN [9].

HIV_NL4-3_ capture was assessed pre-incubating 2.5–3×10^5^ distinct monocyte-derived DCs or blood myeloid DCs at 16°C for 30 min with 10 µg/ml of the α-Siglec-1 mAb 7D2, the isotype control, or 500 µg/ml of mannan. Subsequently, DCs were pulsed with HIV_NL4-3_ at an MOI of 0.1 (50–80 ng of p24^Gag^ estimated by ELISA) for 5 h at 37°C. In parallel, untreated DCs equally pulsed with HIV_NL4-3_ were exposed to inhibitors right after viral capture. After extensive washing, cells were lysed with 0.5% Triton X-100 to measure p24^Gag^ antigen content by ELISA. HIV_NL4-3_ binding was performed pre-incubating LPS mDCs with the indicated mAbs, but maintaining cells at 4°C during viral pulse. Cells were lysed to detect p24^Gag^ or stained with Siglec-1-Alexa 488 7–239 mAb (Ab Serotec) to confirm arrested endocytosis of Siglec-1 at 4°C as compared to cells exposed to the virus at 37°C by FACS.

To assess whether Siglec-1 traffics to the same compartment as sialyllactose-containing vesicles, we adapted our previously described method [13]. Briefly, LPS mDCs were incubated with GM1-LUV_HIV-tRed_, VLP_HIV-Gag-Cherry_, HIV_NL4-3-Cherry_, or HIV_NL4-3_ for 4 h as described above. When indicated, α-HLA-DR-Alexa 647 (Clone L243, Biolegend) was used to reveal LPS mDC membranes. Cells were then fixed, permeabilized, and labeled with Siglec-1-Alexa 488 7–239 mAb. HIV-1 was revealed with α-p24^Gag^-PE (Clone RD1, Coulter). To identify the cytoplasm of pulsed LPS mDCs, some permeabilized cells were also labeled with CellMask Deep Red (Molecular Probes). To detect trafficking of Siglec-1, LPS mDCs were pre-incubated with 10 µg/ml of the mAb 7D2 30 min at 16°C, revealed with a secondary Alexa 488 goat α-mouse IgG mAb (Molecular Probes), washed, and incubated 4 h at 37°C. To determine whether Siglec-1 redistributes to the infectious synapse, LPS mDCs previously pulsed with HIV_NL4-3-Cherry_ for 4 h, extensively washed, and co-cultured with autologous or heterologous CD4^+^ T cells for an additional 2 h were stained with α-CD4-Alexa 647 (Clone OKT4, Biolegend), fixed, permeabilized, and labeled with α-Siglec-1-Alexa 488 7–239. Confocal acquisition and analysis was performed as in [13].

### *Trans*-Infection Assays

DCs were treated and pulsed with HIV_NL4-3_ as described above. After extensive washing, cells were co-cultured with the TZM-bl CD4^+^ target cell line to measure *trans*-infection. Pulsed monocyte-derived DCs or myeloid DCs were co-cultured in quadruplicate or duplicate at a ratio of 1∶1 or 5∶1, respectively. Cells were assayed for luciferase activity 48 h later (BrightGlo Luciferase System; Promega) in a Fluoroskan Ascent FL luminometer (Thermo Labsystems). Background values consisting of non-HIV-1-pulsed co-cultures or reporter CD4^+^ cells alone were subtracted for each sample. To detect possible productive infection of pulsed cells or re-infection events, some DCs were cocultured in the presence of 0.5 µM of the protease inhibitor Saquinavir.

### Transduction of DCs

VSV-G-Pseudotyped SIV3 lentivector (kindly provided by A. Cimarelli) was produced as in [43]. Isolated monocytes (5×10^5^) were infected with SIV3 particles and transduced with two different *SIGLEC1*-specific or one nontarget shRNA control MISSION Lentiviral Transduction Particles (Sigma-Aldrich) at an MOI = 50. Transduced monocytes were differentiated into LPS mDCs and assessed for VLP capture and HIV-1 *trans*-infection as described above. Adequate phenotypic maturation of DCs was evaluated as in [9]. Lentiviral transduction particles carrying the GFP reporter gene cloned in the same pLKO.1-puro vector backbone (MISSION TurboGFP Control Transduction Particles) were used to evaluate transduction efficiency by FACS (estimated 75%–98% at day 7, when cells were employed).

### Transfection of Siglec Constructs

Raji cells (2×10^6^) were transfected with vector backbone pCMV6-Entry (Origene) comprising the coding region of Siglec-1, Siglec-5, or Siglec-7 using Amaxa nucleofector as recommended by the manufacturer. At 36 h posttransfection, cells were assessed for VLP capture and HIV-1 *trans*-infection (at a ratio 2∶1) as described above. When indicated, cells were pre-incubated with decreasing concentrations of 3′-Sialyllactose (Carbosynth) or Lactose (Sigma-Aldrich) 30 min prior to VLP pulse. In experiments with envelope-deficient viruses, 5×10^5^ cells were pulsed with 100 ng of p24^Gag^ estimated by ELISA for 4 h at 37°C and assessed for capture and *trans*-infection (at a ratio 2∶1) as aforementioned. HEK-293T cells were transfected using Fugene HD (Promega) and assessed 24 h posttransfection as described for Raji cells. *Trans*-infection of HEK-293T was tested in a different luminometer (Luminoskan Ascent, Thermo Labsystems), and collected data were normalized to 100%. Transfection efficiency in both cell types was assessed staining cells with α-Siglec-1-PE 7–239 mAb, α-Siglec-7-PE 5–386 mAb (AbD Serotec), and α-Siglec-5/14-PE 1A5 mAb (Biolegend) and evaluated by FACS. Stable Raji DC-SIGN cells were labeled with α-DC-SIGN-PE DCN46 mAb (BD Pharmigen).

### Statistical Analysis

Statistics were performed using paired *t* test (considered significant at *p*≤0.01) or Spearman correlation with GraphPad Prism v.5 software.

## Supporting Information

Figure S1 Blocking effect of α-Siglec-1 mAb 7–239. (A) Capture of VLP_HIV-Gag-eGFP_ by LPS mDCs that had been pre-incubated with decreasing concentrations of α-Siglec-1 mAb 7–239 before VLP exposure for 30 min at 37°C. Data show mean values and SEMs from four donors. (B) Capture of VLP_HIV-Gag-eGFP_ by LPS mDCs that had been pre-incubated with 10 µg/ml of the indicated mAbs before VLP exposure for 3 h at 37°C. Data show mean values and SEMs from two experiments including cells from seven donors.
(EPS)

Figure S2 Geometric MFI of Siglec-1 in LPS mDCs capturing VLP_HIV-Gag-eGFP_. Dot plot shows the gates used to select the 20% of VLP positive cells that capture the lowest and the highest amount of VLPs, respectively. The graph on the right represents the geometric MFI of Siglec-1 within those 20% of the GFP-positive cells that capture the lowest and highest amounts of VLPs, respectively. Data show mean values and SEMs from two experiments including cells from six donors.
(EPS)

Figure S3 Binding of HIV-1 to LPS mDCs at 4°C. (A) Cells were pre-incubated with 10 µg/ml of the indicated mAbs for 30 min, cultured with HIV-1 at 37°C (in red) or 4°C (in blue), washed, and lysed to measure p24^Gag^ by ELISA. Data show mean values and SEMs from two experiments including cells from at least four donors. (B) (Histogram) Representative FACS profile of mock-treated LPS mDCs pulsed with HIV-1 at 4°C (in blue) and surface-stained with α-Siglec-1-Alexa 488 7–239 to confirm temperature-arrested endocytosis of Siglec-1 as compared to cells exposed to the virus at 37°C (in red). (Image) Confocal plane showing surface staining of Siglec-1 in LPS mDCs pulsed with HIV-1 at 4°C (scale bar: 5 µm).
(EPS)

Figure S4 Siglec-1 staining in blood myeloid DCs. (Histograms) Representative cell surface expression profiles of Siglec-1 in blood myeloid DCs that were exposed or not to LPS and analyzed by FACS. (Right graph) Geometric MFI of Siglec-1 labeling. Data show mean values and SEM from cells derived from six donors.
(EPS)

Figure S5 Confocal microscopy analysis of LPS mDCs pulsed with HIV-1. (A) Cells were labeled with HLA-DR-Alexa 647 to detect the plasma membrane and then permeabilized and stained with α-p24^Gag^-PE and α-Siglec-1-Alexa 488 7–239 mAbs. Left image shows the merge of the bright field and the fluorescence of an x-y plane (scale bar: 5 µm). Right image displays the maximum fluorescence intensity of the sac-like compartment where HIV-1 and Siglec-1 accumulate in a 3D volumetric x-y-z data field. (B) Cells were stained for HIV-1 and Siglec-1 as in (A), but cytoplasm was revealed with CellMask after cell permeabilization. Images show the maximum fluorescence intensity of the sac-like compartment where HIV-1 and Siglec-1 accumulate in a 3D volumetric x-y-z data field.
(EPS)

Figure S6 Transfection of Siglecs in antigen-presenting cell lines. (A) (Left histograms) Electroporation induces expression of Siglec-1 in the THP-1 monocytic cell line nonspecifically, but it does not induce it in Raji B cells. Representative overlay profiles of Siglec-1 staining in THP-1 or Raji cells 24 h postelectroporation with different plasmids or mock transfected. (Right histograms) Representative overlay profiles of Siglec-1 staining in THP-1 or Raji cells 24 h after stimulation with LPS. (B) Representative transfection efficiencies of Raji cells assessed by surface expression of distinct Siglecs by FACS. (Left graph) Percentage of positive cells 36 h posttransfection. Data show mean values and SEM from four transfections. (Histograms) Representative cell surface expression profiles of Siglec-1, Siglec-5/14, and Siglec-7 in transfected Raji cells compared to mock-transfected cells. Note that Raji cells express endogenous basal levels of Siglec-5/14.
(EPS)

Figure S7 Blocking effect of sialyllactose. Capture of VLP_HIV-Gag-eGFP_ by Raji cells transfected with Siglec-1 expression plasmid or mock transfected. Cells were pre-incubated with the indicated concentrations of siallyllactose or lactose and exposed to VLPs. Data show mean values and SEMs from triplicates of a transfection.
(EPS)

Figure S8 Transfection of Siglecs in HEK-293T cells. (A) Representative transfection efficiencies of HEK-293T cells assessed by surface expression of distinct Siglecs by FACS. (Left graph) Percentage of positive cells 24 h posttransfection. (Right graph) Geometric MFI values 24 h posttransfection. Data show mean values and SEMs from six transfections. (B) (Graph) Capture of VLP_HIV-Gag-eGFP_ by HEK-293T cells transfected with the indicated expression plasmids for Siglecs or mock transfected. Transfected HEK-293T were pre-incubated with 10 µg/ml of the indicated mAbs and exposed to VLPs. Data show mean values and SEMs from four experiments including cells from eight transfections. (Dot plots) Representative dot plots from Siglec-1 or Siglec-5 transfected HEK-293T pre-incubated with the indicated mAbs and subjected to VLP capture. (Image) Isosurface representation of DAPI-stained nuclei and maximum fluorescence intensity of VLP_HIV-Gag-eGFP_ and Siglec-1 (red staining) in the membrane of a Siglec-1^+^ HEK-293T. (C) Relative HIV-1 transmission from HEK-293T cells transfected with the indicated expression plasmids for Siglecs to reporter CD4^+^ cells. Transfected cells were pre-incubated with the indicated mAbs as in (B) and then exposed to HIV-1. Values are normalized to the level of viral transmission of mock-treated Siglec-1 transfected cells (set at 100% and equivalent to a mean of 0.88 RLU). Data show mean values and SEMs from three experiments including cells from six transfections.
(EPS)

Figure S9 Envelope glycoprotein independent viral capture of Siglec-1 transfected Raji cells. (A) Blue histograms show representative surface expression profiles of Siglec-1 transfected Raji cells and stable Raji DC-SIGN cells labeled with the indicated mAbs. Grey histograms depict isotype controls. (B) Capture of HIV-1 with or without envelope glycoproteins by Raji cells, Raji cells transfected with the expression plasmid coding for Siglec-1, or Raji cells expressing DC-SIGN. Data show mean values and SEMs from two experiments including cells from four transfections. (C) Relative HIV-1 transmission of viruses captured in (B) to reporter CD4^+^ cells. Data show mean values and SEM from two experiments including cells from four transfections.
(EPS)

Movie S1 Confocal microscopy analysis of an LPS mDC pulsed with GM1 containing LUV_HIV-tRed_ and then labeled with Siglec-1-Alexa 488 7–239 mAb. Movie shows 3D reconstruction of the maximum intensity fluorescence of the x-y sections collected throughout the whole cell z-volume every 0.1 µm. Isosurface representation of DAPI-stained nucleus is depicted.
(MOV)

Movie S2 Confocal microscopy analysis of an LPS mDC pulsed with VLP_HIV-Gag-Cherry_ and then labeled with Siglec-1-Alexa 488 7–239 mAb. Movie shows 3D reconstruction of the maximum intensity fluorescence of the x-y sections collected throughout the whole cell z-volume every 0.1 µm. Isosurface representation of DAPI-stained nucleus is depicted.
(MOV)

Movie S3 Confocal microscopy analysis of an LPS mDC pulsed with HIV-1_Cherry_ and then labeled with Siglec-1-Alexa 488 7–239 mAb. Movie shows 3D reconstruction of the maximum intensity fluorescence of the x-y sections collected throughout the whole cell z-volume every 0.1 µm. Isosurface representation of DAPI-stained nucleus is depicted.
(MOV)

## Abbreviations

Ab: antibody
ABS: antibody binding site
DC: dendritic cell
Fab: fragment antigen-binding
FACS: fluorescence-activated cell sorting
eGFP: enhanced green fluorescent protein
FBS: fetal bovine serum
iDC: immature dendritic cell
IL: interleukin
ITIP mDC: clinical grade cocktail-matured dendritic cell
IU: international unit
LPS: lipopolysaccharide
LPS mDC: lipopolysaccharide-matured dendritic cell
LUV: large unilamellar vesicle
mAb: monoclonal antibody
mDC: mature dendritic cell
MFI: mean fluorescence intensity
MOI: multiplicity of infection
PBMC: peripheral blood mononuclear cell
PGE2: prostaglandin E2
qRT-PCR: quantitative real-time polymerase chain reaction
RLU: relative light unit
SEM: standard error of the mean
tRed: Texas red
TNF: tumor necrosis factor
VLP: virus-like particle
VSV: vesicular stomatitis virus
